# Editorial: Neuronal Calcium Sensors in Health and Disease

**DOI:** 10.3389/fnmol.2019.00278

**Published:** 2019-11-15

**Authors:** Daniele Dell'Orco, Karl-Wilhelm Koch, Michael R. Kreutz, Jose R. Naranjo, Beat Schwaller

**Affiliations:** ^1^Section of Biological Chemistry, Department of Neurosciences, Biomedicine and Movement Sciences, University of Verona, Verona, Italy; ^2^Department of Neuroscience, School of Medicine and Health Sciences, University of Oldenburg, Oldenburg, Germany; ^3^RG Neuroplasticity, Leibniz Institute for Neurobiology, Magdeburg, Germany; ^4^Spanish Network for Biomedical Research in Neurodegenerative Diseases (CIBERNED), Instituto de Salud Carlos III, Madrid, Spain; ^5^National Biotechnology Center, CNB-CSIC, Madrid, Spain; ^6^Département des neurosciences et des sciences du mouvement (NMS), Université de Fribourg, Fribourg, Switzerland

**Keywords:** neuronal Ca^2+^ sensors, calcium signaling, molecular basis of disease, calcium bindind proteins, neurological disorders

The precise detection and regulation of free intracellular Ca^2+^ is a prerequisite for keeping normal cell function. Several physiological processes such as fertilization, apoptosis, muscle contraction, neuronal activity, and sensory perception are based on the spatial and temporal variation in intracellular Ca^2+^ and rely on a class of proteins that specifically respond to these highly dynamic stimuli. Neuronal calcium sensor (NCS) proteins are exclusively expressed or enriched in neurons, and their structural and biochemical diversity reflects the multiplicity of their biological roles, which include control of gene transcription, neuronal growth and survival, channel and receptor regulation, neurotransmitter release, synaptic plasticity, and regulation of enzymatic activity. Neurological disorders and neurodegenerative diseases are increasingly associated with altered functions of specific NCS proteins. This Research Topic includes original articles and reviews that provide an interdisciplinary collection of up-to-date biochemical and biophysical research on NCS proteins and their established and novel biological roles in normal and altered (pathological) conditions.

Neuronal calcium sensor-1 (NCS-1) is highly conserved from yeast to human, and it has been implicated in a number of psychiatric conditions including autism, bipolar disorder, schizophrenia, and X-linked mental retardation. At odds with other members of the NCS family, NCS-1 interacts with several cellular targets, which is reflected by a variety of roles. Ratai et al. show that NCS-1 plays an important role in adipocyte function and its deficiency gives rise to obesity and diabetes type 2 in adult mice, thus suggesting a potential genetic link between psychiatric disorders and the risk of being obese. The progressive degeneration of dopaminergic neurons within the *Substantia nigra* is the hallmark of Parkinson's disease and causes its motor symptoms. The link between dopamine release and NCS-1 and its possible implications in Parkinson's disease has been reviewed by Catoni et al., who summarize the role of the interplay between Ca^2+^ and dopamine signaling in neuronal activity and cell death. Simons et al. provide novel data at the transcript level that link NCS-1 deficiency to impaired ATP-production and elevated metabolic stress in *Substantia nigra* dopaminergic neurons in mice. An overview of other novel roles of NCS-1 is provided by the review of Nakamura et al., which focuses on both the neuronal system and the heart, presenting NCS-1 as a regulator of voltage-gated Ca^2+^ channels, ionotropic dopamine receptors, and inositol 1,4,5-trisphosphate receptors. A complex interplay between Mg^2+^, Ca^2+^ and Zn^2+^ binding to NCS-1 leads to the appearance of multiple protein conformations and modulate its functional status as suggested by Tsvetkov et al., who demonstrate that NCS-1 binds Zn^2+^ with differential affinities favoring either the interaction with targets or protein aggregation. Choudhary et al., who focus on optical tweezers investigations to reveal a complex folding mechanism underlaid by a rugged and multidimensional energy landscape, provide insight into the structural and mechanistic details of the folding and misfolding processes of NCS-1 at the single molecule level.

The importance of allosteric interactions between Ca^2+^-binding motifs and amino acids involved in target recognition has been investigated by Marino and Dell'Orco, who suggest for NCS-1, recoverin and GCAP1 an evolution-driven correlation between the amino acids mediating many persistent interactions and their conservation. An inter-domain interaction triggered by Mg^2+^-binding is essential for the ubiquitous Ca^2+^ sensor CIB2 to reach a fully functional conformation, as shown by Vallone et al., who found that the apparently conservative E64D mutation associated with Usher Syndrome 1J and non-syndromic hearing loss prevents this long-range allosteric mechanism. Mutations in calmodulin, another ubiquitous Ca^2+^ sensor, were long thought to be incompatible with life due to the completely conserved amino acid sequence across all vertebrates. The review by Jensen et al. provides an overview of the human missense mutations found in patients with severe cardiac arrhythmias.

Many NCS proteins form dimers, a process that is often Ca^2+^ dependent. The review by Ames summarizes the results of recent studies on GCAPs, VILIP1 and recoverin dimerization. A paradigmatic example of the myristoyl-switch protein, recoverin is involved in the regulation of the phototransduction cascade in rods and cones as reviewed by Zang and Neuhauss. Four different recoverin isoforms exist in zebrafish photoreceptors. Their specific Ca^2+^-sensing properties and conformational changes have been investigated by Elbers et al., who found that binding of Ca^2+^ leads to less pronounced structural rearrangements compared to the bovine ortholog indicating either a modified Ca^2+^-myristoyl switch or no switch at all. Novel roles for recoverin in health and disease-associated conditions have been found by Zernii et al. who provide *in vitro* and *in vivo* evidence that illumination of the mammalian retina leads to the accumulation of disulfide dimers of recoverin, which are thought to favor light-induced oxidative stress and photoreceptor apoptosis.

The complex between GCAPs and the retinal guanlylate cyclases is a crucial component of the vertebrate phototranduction machinery as it regulates the interplay between the second messengers cGMP and Ca^2+^. Rehkamp et al. present a chemical cross-link/mass spectrometry investigation on the interaction between GCAP2 and GC-E, while Wimberg et al. investigate five recently identified GC-E mutants associated with Leber Congenital Amaurosis, a cone-rod dystrophy, finding severe alteration of the cGMP synthesis. Another prototypical guanylate cyclase, the atrial natriuretic factor receptor guanylate cyclase is found to be regulated by the Ca^2+^ sensor neurocalcin δ and hormone ANF via two distinct and non-overlapping transduction modes, as elucidated by Duda et al.

Ca^2+^-binding proteins have been found to be involved in the complex etiology of psychiatric disorders. By combining a stereology-based approach and molecular analyses Lauber et al. investigated the involvement of parvalbumin in autism spectrum disorder, finding a dysregulation of its expression in *Cntnap2* knockout mouse. The review by Mundhenk et al. presents a detailed structural and biophysical characterization of the Ca^2+^-sensor proteins caldendrin and calneuron-1 and−2, focusing also on their cellular function and their role in neuropsychiatric disorders.

A role for the multi C_2_-domain protein otoferlin in modulating the Ca^2+^-triggered exocytosis at the ribbon synapse in mouse inner hair cells is proposed by Takago et al. by a combination of electrophysiology and biochemical analyses. The importance of Ca^2+^-binding proteins in regulating the fundamental process of exocytosis and synaptic coupling is emphasized in the review by Maj et al., focusing on secretagogin and its novel roles in developing and adult neuronal cells and Bornschein and Schmidt, focused on synaptotagmin 1 and 2 and their role in presynaptic voltage-gated Ca^2+^ channel regulation.

Downstream Regulatory Element Antagonist Modulator (DREAM)/KChIP3 exerts multiple functions, including the regulation of A-type outward potassium currents. The contribution by Peraza et al. identifies by biochemical and biophysical investigations a novel ligand of DREAM that modulates Kv4 potassium channels currents, with consequences that are relevant for physiology and disease. The role of DREAM/KChIP3 in pain transmission and its possible involvement in emotional processing was studied by Guo et al., who assess the pain sensitivity and negative emotional behaviors of Kcnip3^−/−^ rats and find a possible role for the protein in central nociceptive processing. Novel tools to regulate the role of DREAM in the endoproteolysis of endogenous presenilin-2 in mouse brain are presented by Naranjo et al., who suggest that the interaction between the two proteins may have a therapeutic potential in Alzheimer's disease. Finally, the review by Néant et al. summarizes the current knowledge regarding Ca^2+^ signaling in quiescent glioblastoma stem-like cells and discussed how Ca^2+^ via KCNIP proteins may affect gene expression in glioblastoma.

Taken together, this Research Topic delivers new visions to our knowledge on NCS proteins and will stimulate future research. We wish to thank all the authors for having submitted papers of high quality and all the reviewers that contributed with constructive and fruitful suggestions.

## Author Contributions

All authors listed have made a substantial, direct and intellectual contribution to the work, and approved it for publication.

### Conflict of Interest

The authors declare that the research was conducted in the absence of any commercial or financial relationships that could be construed as a potential conflict of interest.

